# Review of Crystalline Structures of Some Selected Homologous Series of Rod-Like Molecules Capable of Forming Liquid Crystalline Phases

**DOI:** 10.3390/ijms12117360

**Published:** 2011-10-28

**Authors:** Peter Zugenmaier

**Affiliations:** Institute of Physical Chemistry, TU Clausthal, Arnold-Sommerfeld-Str. 4, D-38678 Clausthal-Zellerfeld, Germany; E-Mail: Zugenmaier@pc.tu-clausthal.de; Tel.: +49-0-5323-72-2584; Fax: +49-0-5323-72-4835

**Keywords:** crystal structure of 4 homologous series, conformation and packing arrangement, unconventional packing arrangements

## Abstract

The crystal structures of four homologous series of rod-like molecules are reviewed, two of which form hydrogen bonds and two with a symmetric chemical constitution. Many of the compounds investigated turn into liquid crystalline phases upon temperature increase. It is of valuable interest to know possible conformations and possible packing arrangements as prerequisites to model liquid crystalline structures. The hydrogen bonds of homologous series of pure 4-(ω-hydroxyalkyloxy)-4′-hydroxybiphenyl (HnHBP, *n* the alkyloxy tail length) are realized through head to tail arrangements of the hydroxyl groups and crystallize except one compound in chiral space groups without the molecules containing any asymmetric carbon. The hydrogen bonds of the homologous series of 4-substituted benzoic acids with various lengths of the tail provide dimers through strong polar bonding of adjacent carboxyl groups and thus provide the stiff part of a mesogenic unit prerequisite for liquid crystalline phases. The homologous series of dialkanoyloxybiphenyls (BP-*n*, *n* = 1, 19), of which nine compounds could be crystallized, show liquid crystalline behavior for longer alkane chain lengths, despite the high mobility of the alkane chain ends already detectable in the crystal phase. A single molecule, half a molecule or two half molecules form the asymmetric unit in a centrosymmetric space group. The homologous series of 1,4-terephthalidene-bis-*N*-(4′-*n*-alkylaniline) (TBAA-*n*) exhibit a large variety of packing arrangements in the crystalline state, with or without relying on the symmetry center within the molecules.

## 1. Introduction

In this review, the crystal structures determined by single crystal X-ray evaluation are presented and compared for four selected homologous series at ambient temperature. For two series of compounds, hydrogen bonding plays an important role and their influence on packing arrangements and symmetry of space groups will be considered. The other two series possess symmetrical chemical constitutions and can provide an inversion center within each of the molecules, which may or may not influence the arrangement of molecules in the crystal structure. Many of the crystal structure data are published here for the first time, others are provided in the literature [1–6] and further structures are represented by models [7–10]. These crystallographic data provide valuable support for explaining features of the phase behavior including liquid crystals as demonstrated by Doucet *et al*. [11] or by Heiske [8], Thyen [9] and Roman *et al*. [10]. Doucet *et al*. determined the crystal structure of a single compound in a first step and then applied these findings for modeling a smectic phase in a succeeding step.

Studying homologous series provides important information about possible changes of the conformation and packing arrangement of molecules by a variation of the molecular length. Such structural changes are caused by the influence of the interactions between molecules and may lead to unconventional packing of the molecules or symmetry changes by the space group to be considered for evaluating structural models. Not all members of a homologous series provide sufficient high-quality crystals for single crystal structure studies and are left out in this review. Almost all crystallographic structures provided in this review have been re-determined with improved data and programming. A prediction of possible liquid crystalline phases has so far not been achieved and is not intended as a goal in this work, rather, basis crystallographic data are provided for modeling structures at higher temperatures.

**Scheme 1:**
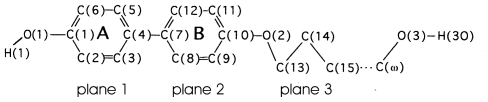
Representation of the homologous series of 4-(ω-hydroxyalkyloxy)-4′-hydroxybiphenyl abbreviated HnHBP.

**Scheme 2:**
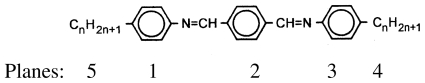
Representation of the homologous series of 1,4-terephthalylidene-bis-*N*-(4′-*n*-alkylaniline) abbreviated TBAA-*n*.

**Scheme 3:**
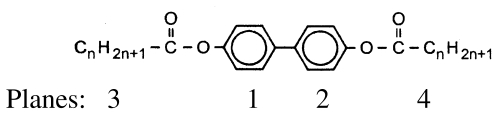
Representation of the homologous series of 1,4-di-*n*-alkanoyloxy-biphenyl abbreviated BP-*n*.

The following homologous series of compounds were investigated (Scheme 1–3):

## 2. Discussion of the Crystallographic Structures

### 2.1. 4-(ω-Hydroxyalkyloxy)-4′-hydroxybiphenyl HnHBP

Single crystals of sufficient quality have been obtained by dissolving the compounds in a suitable solvent, mostly butyl acetate, and succeeding slow evaporation of the solvent at room temperature. The synthesis of the compounds and the phase behavior are described in the doctoral thesis of Heiske [8]. Liquid crystalline phases of the smectic type are only observed by cooling from the melt.

**Figure 1:**
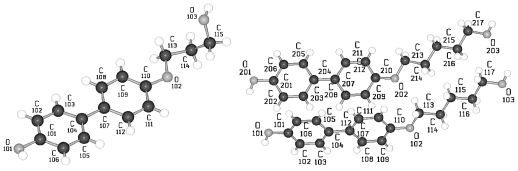

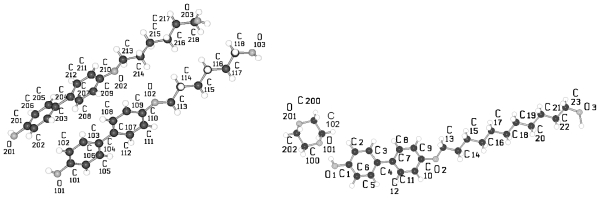
Representation of the asymmetric unit (molecules) and numbering of atoms for the homologous series HnHBP, here shown for H3HBP, H5HBP, H6HBP (valid also for HnHBP, *n* = even), and H11HBP complexed with 1,4-dioxane as representatives for various crystal structures.

The main feature of the homologous series of HnHBP consists of a hydrogen bond network between the oxygen of the hydroxy head group O1 and the hydroxy group at the tail end O3 (*cf.* Figure 1). This kind of network is formed either by polar parallel packing of the molecules in the chiral space group P2_1_ (*n* = 4, 6, 8, 10) with two molecules in the asymmetric unit or by antiparallel packing in the chiral (or also called Sohncke) space group P2_1_2_1_2_1_ (*n* = 3) with one molecule as asymmetric unit but also in the centrosymmetric space group P2_1_/c (*n* = 5), here with pairs of molecules as asymmetric unit (Figure 1). An exception represents the solvent complex of H11HBP with 1,4-dioxane. The oxygen of the dioxane molecule is involved in a hydrogen bond with the oxygen of the hydroxy head of H11HBP (Figure 1) and leaves the oxygen of the tails to form a network of hydrogen bonds. A summary of the crystallographic data is listed for all HnHBP in Table 1.

The conformations of the two molecules in the asymmetric unit of the homologous compounds investigated (*n* = 4–10; Figure 1) are very similar as verified by the twisting of the three planes (Table 2), which are introduced in the schematic representation of HnHBP above (Scheme 1), despite the fact that various packing arrangements are present leading to different space groups. However, different conformations occur at the hydroxy tail end of these two molecules and lead to the conclusion that these two molecules cannot be related by any symmetry element.

**Figure 2:**
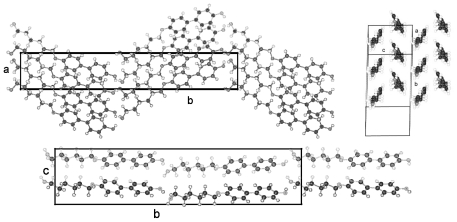
Representation of the packing arrangement of H6HBP, space group P2_1_, projected in [001] and [100] direction and a projection along the molecular axes of one monolayer showing the differently oriented two molecules of the asymmetric unit. Similar patterns can be drawn for all HnHBP in space group P2_1_.

The structures of HnHBP with *n* = 4, 6, 8, 10 belong to the polar Sohncke space group P2_1_, unusual for molecules without asymmetric carbon atoms; with *n* = 5 to the centrosymmetric space group P2_1_/c as well as the solvent complex with *n* = 11 to the centrosymmetric space group C2/c but with *n* = 3 to the Sohncke space group P2_1_2_1_2_1_. The structures belonging to space group P2_1_ show two different inclinations of the parallel running molecular axes of succeeding two layers in projection on the a,b-plane (Figure 2), which results in a shorter b axis as expected from the length of the molecules. On the b,c-plane the molecules show almost a parallel arrangement with the b-axis (Figure 2). The shortening of the b-axis amounts to (37.58−34.52) = 3.1 Å for H6HBP. The overall structure can be described as a fishbone pattern with two adjacent monolayers repeated periodically in b-direction. In each monolayer the parallel running molecules (represented e.g. by the biphenyl groups) are twisted in different directions as shown in Figure 2 in a projection down the molecular axes. This projection also shows that the molecules form strings, which can be arranged in planes. The hydrogen bonding network is established between the hydroxy head group O101 and the hydroxy tail group O203′ or O201 and O103′ as well as the tail end O103 and the hydroxy head O101′ or O203 and O201′, respectively. The possible hydrogen bonds for all HnHBP are listed in Table 3.

**Figure 3:**
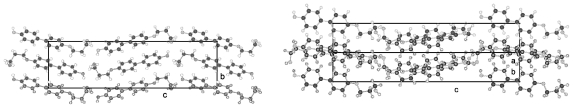
Representation of the antiparallel arrangement of H3HBP in space group P2_1_2_1_2_1_ projected along [100] and along the diagonal of the a,b-plane.

The molecules of H3HBP crystallize in space group P2_1_2_1_2_1_ with only one molecule as asymmetric unit and exhibit antiparallel packing represented in Figure 3. Despite antiparallel packing, the hydrogen bonds are still formed between the head group O101 and the tail group O103 and *vice versa* between O103 and O101, as listed in Table 3. The molecules show a slight fishbone pattern that resembles the periodically arranged monolayer structures of space group P2_1_ but is less expressed than by H6HBP in Figure 2.

**Figure 4:**
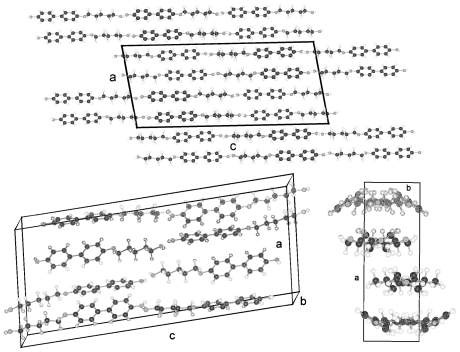
Antiparallel arrangement of the molecules of H5HBP in the centrosymmetric space group P2_1_/c projected along [010], inclined to a small extent from this projection and along [001] shown at the bottom right corner.

In contrast, the compound of H5HBP forms a centrosymmetric packing arrangement in space group P2_1_/c. Two molecules form the asymmetric unit and allow again a head to tail hydrogen bonding system (*cf.* Table 3), here within a single molecular string and no cross over between the two molecules in the asymmetric unit as clearly shown in Figure 4. All the molecules crystallizing in space group P2_1_ reveal a cross over within the hydrogen bonding network of the head and tail ends between the two molecules in the asymmetric unit (Table 3). Different slight tilts of neighboring molecules are present in H5HBP and shown in the [001] projection in Figure 4. Small differences in the hydrogen bonding systems are expressed in the strength of the interactions and can be demonstrated by the FTIR technique as shown below in Figure 7 and discussed later. These tracings were recorded with crystalline samples produced by dropping an acetone solution on KBr tablets.

**Figure 5:**
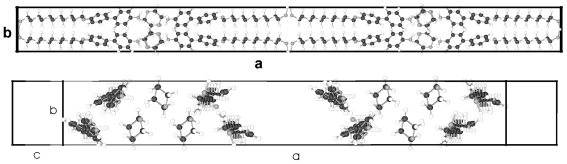
Representation of packing arrangement of H11HBP complexed with dioxane in space group C2/c projected along [001] and in projection along the molecular axes to show the strong tilt of the molecules.

**Figure 6:**
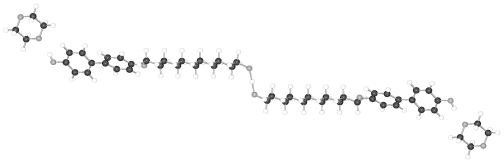
Representation of the head-head and tail-tail arrangement of a string of H11HBP and the hydrogen bonding between the oxygen of the tail groups, as well as between the hydroxy group of the head and the 1,4-dioxane.

**Figure 7:**
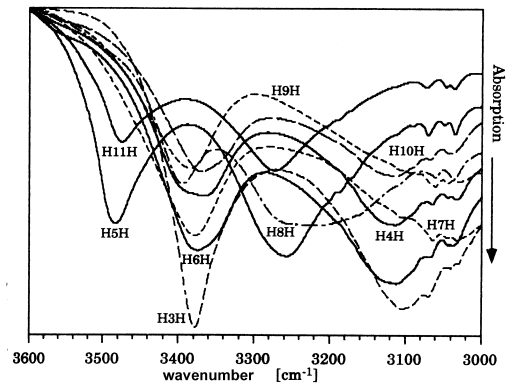
FTIR spectra of the homologous series of HnHBP, here abbreviated HnH [8].

H11HBP complexed with 1,4-dioxane, which is part of the crystal structure, is the only compound showing tail-to-tail hydrogen bonding (Figure 5). This kind of arrangement is possible since the oxygen of the head group forms a hydrogen bond with the oxygen of the dioxane molecule and thus allows tail-to-tail hydrogen bonding. A continuous string of succeeding molecules exhibits periodical head-head and tail-tail arrangement. A reversal of the molecular direction occurs within a string and therefore, a molecular direction cannot be defined (Figure 6). The structure of the H11HBP complex consists of a single molecule of H11NBP and half a dioxane molecule as asymmetric unit and crystallizes in the centrosymmetric space group C2/c with the inversion center placed in the center of mass of the dioxane molecule. The strength of the hydrogen bonds resembles the ones of H5HBP as deduced from the FTIR measurements in Figure 7.

The crystal structures determined for the HnHBP compounds show some surprising features regardless of a variety of symmetries (space groups) in their packing arrangements. The unit cells of the compounds with even methylene groups in the alkoxy tail resemble each other in the lateral a- and c-dimensions as well as in the monoclinic angle β (close to 90°). The b-dimension mirrors the lengths of the molecules. This is also true for the dimensions of H3HBP exhibiting a one-molecule asymmetric unit with antiparallel packing arrangement in space group P2_1_2_1_2_1_ and β = 90° and also surprisingly holds for H5HBP, if the a-dimension is reduced to ½ due the fact that the number of molecules is doubled as compared to the other compounds discussed. All these molecules form periodic somewhat bended strings with molecular tail to head arrangements except the H11HBP complex.

An absolute configuration of the molecules for the chiral space groups could not be determined. Therefore, both enantiomeric configurations have to be considered as equal probable since the *R*-value and the goodness of fit are the same for both configurations (*cf.* Table 1). H11HBP complexed with 1,4-dioxane represents an exception with tail to tail and head to head arranged molecules and the molecular axes exhibit a strong uniform tilt. Different conformational positions of HnHBP occur for the oxygen of the hydroxy group at the tail end of the molecule. This oxygen lies in plane with the carbons of the methylene chain (all trans conformation) for HnHBP (*n* = 5, 11) in the centrosymmetric space groups, but this conformation deviates from the all trans conformation at least for one of the two molecules forming the asymmetric unit in space group P2_1_. A gauche conformation is present for this oxygen of the chiral space group P2_1_ (*n* = 4, 6, 8, 10) and for the single molecule of the asymmetric unit for space group P2_1_2_1_2_1_ (*n* = 3). The plane 3 through the alkoxy chain deviates little from the plane through the phenyl ring 2 for all HnHBP except for H3HBP (Table 2) for which a twist of 61° is found.

The hydrogen bonds of the crystal structures occur in pairs of different length equivalent to pairs of different strengths or pairs of peaks in the FTIR spectra (Figure 7). All hydroxy groups are involved in hydrogen bonds. The longer intermolecular O..O distances are represented by bands at *ca.* 3380 cm^−1^, the shorter ones by bands at *ca.* 3100 cm^−1^. The longer and comparable O..O distances of H5HBP and H11HBP are shifted to longer wavenumbers and represent weaker hydrogen bonds (Figure 7).

### 2.2. Crystal Structures of Some 4-Substituted Benzoic Acids

A mixed dimer formation and its phase behavior has been investigated in binary systems of 4-substituted benzoic acids and the phase diagrams of two systems with 4-[(*S*)-(−)-2-methylbutoxy]benzoic acid (MBOBA) as one component and 4-(hex-5-enoxy)benzoic acid (HOBA) and 4-(dec-9-enoxy)benzoic acid (DOBA), respectively, as the second component explicitly determined. These mixed dimers form chiral nematic and crystalline phases, and in addition the MBOBA-DOBA system a twisted smectic C-phase. In contrast pure MBOBA only shows crystalline dimers. These findings have been published and the crystalline structures of a homologous series of benzoic acids have been presented by models without detailed evaluation of the single crystal X-ray data [10]. Structural details are now offered and basic crystallographic data are collected in Table 4.

Generally, molecules of benzoic acids pair through strong intermolecular hydrogen bonds by the carboxyl groups and form dimers. A dimer has a larger aspect ratio and twice the number of polarizable aromatic rings and carboxyl groups with respect to a single molecule and the possibility of liquid crystalline behavior is enhanced. A description of the synthesis and characterization of the compounds that are namely 4-(but-3-enoxy)benzoic acid (BOBA), 4-(pent-4-enoxy)benzoic acid (POBA), 4-(dec-9-enoxy)benzoic acid (DOBA), and 4-(undec-10-enoxy)benzoic acid (UOBA) is provided in the cited paper [10]. Here, in addition the crystal structures of 4-(hex-5-enoxy)benzoic acid (HOBA) as well as 4-(prop-2-enoxy)benzoic acid (PROBA) are discussed.

All these compounds are arranged in antiparallel fashion to compensate the strong dipoles of the carboxyl groups of the molecules and all form dimers in the crystalline state. They have in common a similar distance of the two C–O bonds of the carboxyl group of which both oxygens are involved in hydrogen bonds and a gauche position of the alkoxy segment of the linear substituent. A center of inversion symmetry, often observed, is devoid in crystallites existing of a single *S*-configuration for example.

Single crystals of *S*-MBOBA were grown by slow crystallization of dissolved compounds in warm ethanol and slow cooling with evaporation of the solvent. All the other compounds of the homologous series of benzoic acids considered were crystallized by the same procedure. Due to insufficient data, the absolute configuration of the *S*-configured compound could not be determined with single crystal data evaluation and therefore, the *S*-configuration provided by synthesis was assumed. Both forms *S*-MBOBA and the racemic mixture (*R*,*S*-MBOBA) were investigated and show almost identical triclinic unit cells containing two molecules (Table 4).

**Figure 8:**
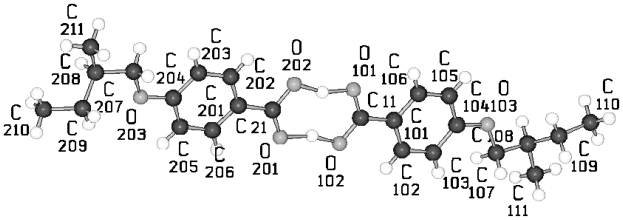
Representation of the conformations and atom numbering of *S*-MBOBA. A similar molecular structure with one molecule in *R*- the other in *S*-configuration occurs in the racemic (*R*,*S*)-MBOBA.

**Figure 9:**
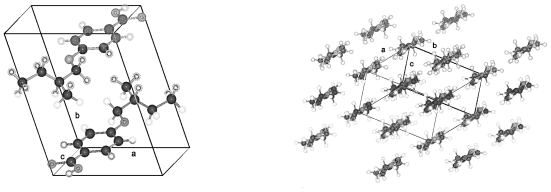
Representation of packing arrangement of a 1:1 racemic mixture of (*R*,*S*)-MBOBA in two projections. The asymmetric unit of space group P-1 contains one molecule, which is related by an inversion center to a second one through strong hydrogen bonds to form a dimer as shown in Figure 8. The projection down the dimeric axes leads to strings arranged in planes.

The structure of *S*-MBOBA in space group P1 consists of two molecules (asymmetric unit) of the same configuration, which were assigned to the *S*-form (Figure 8). The structure of single crystals for a racemic mixture was first determined in space group P1. Here, each of the two molecules in the triclinic unit cell exhibits the expected absolute *R*- and *S*-configuration, respectively. The two molecules are related by inversion symmetry, which allows space group P-1 to be assigned to the racemic (1:1) mixture (Figure 9). The existence of only one crystal appearance of the racemic mixture with two molecules of opposite configuration suggests that the strong hydrogen bonds between the two configurationally different molecules are formed during crystallization. Otherwise crystallites containing only molecules of the *S*- or *R*-configuration should also be observed since in solution, from which the single crystals are grown, all possible combinations of *S*- and *R*-dimers should be present. Crystals of pure *S*- and *R*-dimers are actually absent. The packing density of racemic MBOBA in the centrosymmetric space group P-1 is higher than for *S*-MBOBA in space group P1 and is therefore favoured (*cf.* Table 4a). This result confirms our previous observation of mixtures of *R*- and *S*-benzoic acids with different tail ends that also crystallize in one form only [12].

**Figure 10:**
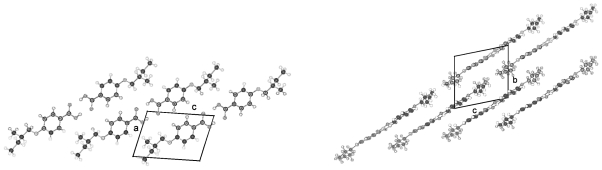
Representation of the packing arrangement of *S*-MBOBA, space group P1, in two projections along [010] and [100] to show the sheet-like structure. A similar representation can be given for the racemic structure of a (1:1) *R*,*S*-mixture.

The packing arrangements of the two structures, the *S*-form and *R*,*S*-(racemic) form are very similar as can be concluded from the almost identical size of the two unit cells (Table 4a). An inversion center of the racemic form lies between the two carboxyl groups and similar C–O bond lengths of 1.27 Å for both C–O distances of the acid group as well as equal O..O distances for the two hydrogen bonds of *ca.* 2.60 Å are detected (*cf.* Figure 8) as expected from published benzoic acid structures. An inversion center is missing in the crystal structure of the *S*-configured molecules in space group P1 (Figures 8,9). Instead a second molecule with the same *S*-configuration is placed almost in the same position as found in the racemic structure and leads to hydrogen bonding without an inversion center in between the two molecules. In an overall point of view it is rather difficult to distinguish between the two models of *S*- or racemic *R*,*S*-configured structures. Only one drawing is represented to avoid duplication and represents as an overall model the other crystal form also. Figures 9 and 10 illustrate different projections of the structure, which can be interpreted that the molecules form strings and are assembled in sheets.

**Figure 11:**
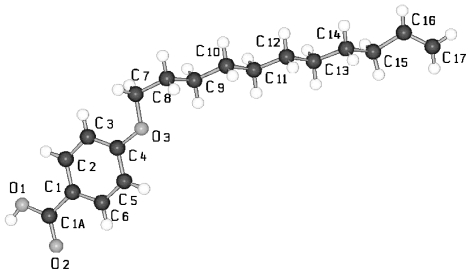
Representation of the conformation of a molecule of 4-(undec-10-enoxy)benzoic acid (UOBA) and numbering of the atoms. (A similar numbering scheme was used for the shorter 4-substituted benzoic acid).

The crystal structures of UOBA, DOBA and HOBA strongly resemble each other and will now be discussed: Figure 11 represents the conformation of UOBA with atomic numbering, which can be applied to all 4-substituted benzoic acid molecules discussed in this review. The phenyl rings of the hydrogen bonded two UOBA molecules lie almost coplanar and the plane through the methylene chain with the slightly bent vinyl tail end is twisted by 68° towards the phenyl planes (Table 5a). The vinyl end groups of the three molecules UBOA, DOBA and HOBA are very mobile and bond lengths and angles are detected out of any standard range if not restrained. The best description of the tail end can be obtained by introducing partial occupancy of at least two rotational positions. This partial occupancy description is omitted in the presentation of conformation and packing arrangement in the figures and only one position is presented to provide a clearer picture. The twist angles of the planes of the phenyl rings towards the alkane tails described by Φ (Table 5a) are close for UOBA, DOBA, HOBA and BOBA. The torsion angles τ1(O3-C7-C8-C9) express the placement of the alkoxy groups and are similar in absolute value for the four compounds (Table 5a). The signs play no role since they are inverted by the inversion symmetry, which correlates two molecules in the unit cell. The torsion angles τn represent the allyl terminal group ending with the double bonded terminal C-atom.

**Figure 12:**
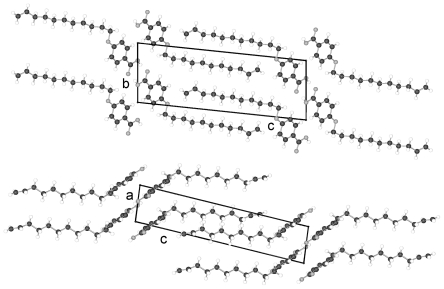
Representation of the packing arrangement in space group P-1 of 4-(undec-10-enoxy)benzoic acid (UOBA) in two projections along [100] and [010].

**Figure 13:**
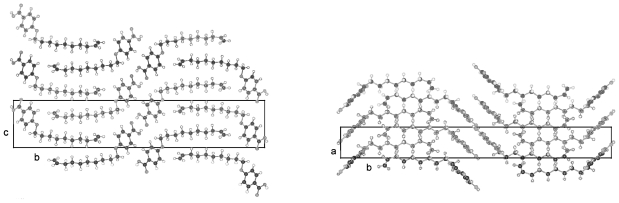
Representation of the packing arrangement in space group P2_1_/n of 4-(dec-9-enoxy)benzoic acid (DOBA) in two projections along [100] and [010].

**Figure 14:**
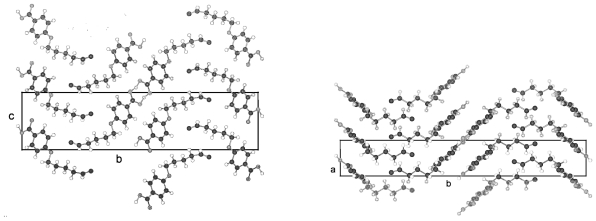
Representation of the packing arrangement in space group P2_1_/n of 4-(hex-5-enoxy)benzoic acid (HOBA) in two projections along [100] and [010].

The packing arrangement of UBOA in space group P-1 is shown in two projections in Figure 12. The phenyl rings of two molecules connected by hydrogen bonding lie almost coplanar and the plane through the methylene chain is in gauche position. These two molecular parts pack in staples of their own, which arrange in two different directions. This type of packing seems to represent preferred arrays for the molecules since this kind of interaction occurs also for DOBA and HOBA (Figures 13 and 14) despite the fact that, these two compounds crystallize in space group P2_1_/n as compared to P-1 for UOBA. The dimensions of the unit cells are nevertheless comparable if a doubling of the c-dimension for the unit cell of UOBA is introduced as well as considering the influence of the elongation of the alkoxy tail (*cf.* Table 4). The gauche conformation between phenyl ring and alkoxy tail hinders a straight molecular string of adjacent succeeding molecules to be formed and causes a break of the sheets containing these two parts of the molecules. Such interdigitated arrangements are found in liquid crystalline phases. Nevertheless, close packing is achieved in space group P-1 as well as in P2_1_/n as concluded from the calculated density of the crystals.

The discussed benzoic acids crystallizing in centrosymmetric space groups with an inversion center between the carboxyl groups form so called dimer molecules and can be compared with the discussed BP-n below. The asymmetric unit consists of half a dimer *i.e*., a single molecule. The sign of the torsion angle τ1 is inverted when the original and the inversion symmetry related molecule is considered as for *R*,*S*-MBOBA in contrast to *S*-MBOBA for which τ1(1) and τ1(2) of the two related molecules have the same sign and almost the same size in both conformations (*cf.* Table 5b).

**Figure 15:**
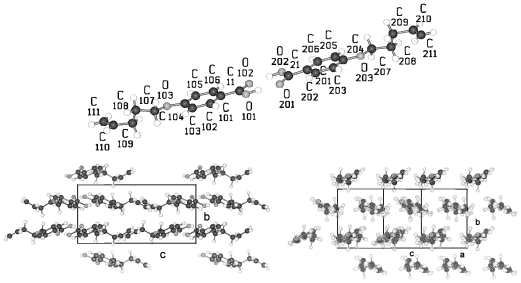
Representation of the conformation of two independent molecules forming dimers and the packing arrangement in space group P2_1_ of 4-(pent-4-enoxy)benzoic acid (POBA) in two projections along [100] and along the molecular axes.

**Figure 16:**
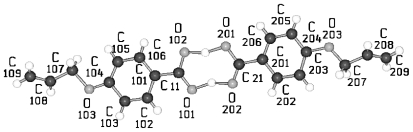

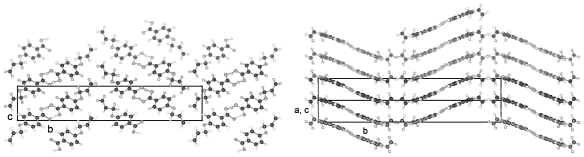
Representation of the conformations of two molecules of PROBA as asymmetric unit with the numbering of the atoms and the projections of the packing arrangement in space group P2_1_ along [100] and the diagonal of the a,c-plane.

However, for POBA, refined in the chiral space group P2_1_, the size of the torsion angles τ1(1) and τ1(2) describing the position of the alkoxy group of the two independent molecules 1 and 2 (*cf.* Table 5b) as well as torsion angles τn(1) and τn(2) for the allyl terminal group almost agree but the signs of τ1(1), τn(1) differ from τ1(2), τn(2), which should not occur for a chiral space group. This inversion of signs for τ1(1) and τ1(2) also holds for PROBA but the values vary to some extent due to broad and less precise determined intensities. This observation supports the idea that the two independent molecules of POBA and PROBA are related by an inversion symmetry element as expressed in the drawing of the conformations in Figures 15 and 16. A transformation of the coordinates with an inversion center between the two carboxyl groups as origin reveals that the two molecules are inversion symmetry related. This inversion symmetry element does not appear in space group P2_1_ and therefore, it seems likely that P2_1_, in which the compounds have been successfully refined, represents a subgroup of a centrosymmetric space group (Table 4b). This space group has yet to be determined; attempts were not successful introducing a monoclinic unit cell with 2_1_-screw axes.

The remaining three crystal structures to be discussed, POBA, BOBA and PROBA, show different conformations and packing arrangements probably due to a diminishing influence of the alkoxy side groups that means the lowest energy state is no longer predominantly determined by an all-trans conformation of the methylene groups, rather than the packing of the molecules essentially contributes to the overall low free energy.

POBA and PROBA crystallize in space group P2_1_ determined by the extinction of reflections of the monoclinic unit cell. This space group is confirmed by the fact that the atoms of two independent molecules are clearly revealed by structure solving procedures and that the crystal structure refinement with two inversion symmetry related molecules results in convincing low *R*-values and goodness of fits.

The conformation of the two independent molecules in the asymmetric unit of POBA is shown in Figure 15. The two molecules form hydrogen bonded dimers. The unit cell contains 2 of these dimers, respective 4 single molecules. Figure 15 also shows the b,c-projection of the packing arrangement of the two dimers in the unit cell with the dimer axes perpendicular to b and a in projection down the molecular axes, which suggests that molecular strings can be defined and that these strings are arranged in planes.

PROBA also crystallizes in the space group P2_1_ of a four molecules containing monoclinic unit cell but the packing arrangement differs considerably from POBA (Figure 16). The unit cell parameter b represents for POBA (Figure 15) the shortest lattice axis, for PROBA the longest (Table 4b) and in addition a and c have almost the same size for PROBA. The b,c-projection represents as well as the b,a-projection (not shown) planes in which the molecular (dimer) strings are located in a fishbone pattern. Rotating the [100] projection around b leads to a view in which the fishbone planes are shown edge on (projection along the diagonal of the a,c-plane), which also exhibits a fishbone pattern. The two projections in Figure 16 resemble very much the ones shown for *S*-MBOBA in Figure 10 in space group P1 with only one dimer in the unit cell leading to a single orientation of the molecules and therefore, no fishbone pattern can occur in both projections in Figure 10.

**Figure 17:**
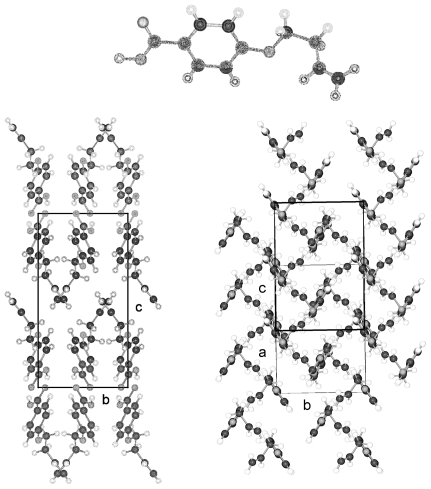
Representation of the conformation and the packing arrangement of 4-(but-3-enoxy)benzoic acid (BOBA), space group P2_1_/a, in two projections along [100] and down the molecular axes.

A disturbed space group P2_1_/a deduced by the extinct reflections can describe the monoclinic crystal structure of BOBA. Due to partial occupancy of the vinyl tail end, subgroup Pa may also be a good choice indicated by the placement of the terminal vinyl carbon in two positions of which one deviates from the required inversion symmetry of space group P2_1_/a (*cf.* Table 5a). Figure 17 shows the conformation and packing arrangement with one partial position of the vinyl tail end. The formation of dimers through hydrogen bonding of the two carboxyl groups seems to dominate the structure and forces the vinyl ends to deviate from the molecular (dimeric) axes. Therefore, the projection down the molecular axes does not provide the clear pattern as often observed in the 4-substituted benzoic acids.

The 4-substituted benzoic acids studied form all hydrogen bonded dimers with almost equal lengths of the two oxygen distances of *ca.* 2.60 Å. The plane of the carboxyl group deviates little from the phenyl ring plane. The inversion center of the centrosymmetric space groups except for the *S*-configured compound coincides with the center of mass of the two carboxylic acid groups. Therefore, the two phenyl rings of the dimer lie almost coplanar. The *S*-configured compound *S*-MBOBA also exhibits hydrogen bonding of the same appearance and strength between the two carboxyl groups and also leads to the formation of dimers.

The 4-substituted benzoic acids crystallizing in centrosymmetric space groups can be regarded as symmetrical dimers and compared with the below discussed dialkanoyloxybiphenyls (BP-*n*) and 1,4-terephthalidene-bis-*N*-(4′-*n*-alkylaniline) (TBAA-*n*), which represent symmetrical molecules. The asymmetric units of these symmetrical molecules consist either of half a molecule or an entire molecule in contrast to the asymmetric units of the dimers, which always represent a whole molecule.

Mixed dimers of 4-substituted benzoic acids provide the possibility to create chiral structures with one component consisting of a chiral compound only, the other of a non-chiral compound. The creation of dimers of various lengths increases the variability of compounds to be investigated especially for liquid crystalline phases and to study their correlation to structural and thermal properties.

### 2.3. 1,4-Di-n-alkanoyloxy-biphenyl (BP-n)

Dialkanoyloxybiphenyls (BP-*n*, *cf.* Scheme 3) with *n* = 1–8, 11 were first synthesized by Pakkal [13]. Thyen [9] then increased the number of available compounds synthesizing BP-*n* from *n* = 1 to 19 and investigated the physical properties including the liquid crystalline phase behavior comprehensively as well as their classification for compounds with *n* ≥ 5, which exhibit highly ordered smectic phases, most probably SmG as high temperature phase. Some selected crystal structures have been determined in his doctoral thesis [9] and in two publications [4,5]. The homologous series investigated may serve as low molecular mass models for liquid crystalline polyester as studied by Krigbaum [14] and provide insights to molecules with a stiff biphenyl center part and flexible aliphatic tail groups.

BP-*n* represent symmetrical molecules by constitution, which may influence the packing arrangements in the crystalline state. An inversion center can occur between the two phenyl rings and half a molecule then represents the asymmetric unit of a centrosymmetric space group. The asymmetric unit may be extended to two half-molecules, which may be linked to two complete single molecules by inversion centers, leading to two conformational different molecules in a two-chain unit cell of space group P-1. It has been also observed that BP-*n* exhibit an individual molecule as asymmetric unit devoid of any symmetry within the molecule and the inversion centers lying between the molecules to result in a centrosymmetric space group as well. In this case the symmetry of the constitution does not play any role. The various symmetries show up in distinct conformations for various BP-*n* and will be discussed.

Single crystal structures of dialkanoyloxybiphenyls (BP-*n* with *n* = 1, 2, 5, 6, 8, 11–14 were grown by slow evaporation of toluene or trichloromethane solutions and their structures are presented below. A summary of the basic crystallographic data is collected in Table 6. All crystalline compounds investigated belong to space group P-1 except BP-2, which crystallizes in P2_1_/c with half a molecule as asymmetric unit. The lattice parameter for BP-*n* with *n* = 6–14 are very similar if the elongation of the c-axis due to the actual length of the molecules is taken into account. Two half-molecules as asymmetric unit describe the structures of these compounds. In contrast the asymmetric unit consists of a single molecule for BP-1 and BP-5.

**Figure 18:**
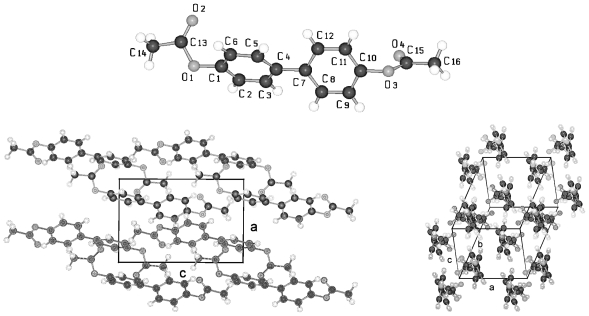
The conformation of BP-1 and the numbering of atoms. The hydrogens carry the same numbers as the carbons to which they are attached. A similar numbering scheme is applied to all BP-*n*. Also represented in this figure is the packing arrangement in projection on the a,c-plane and down the molecular strings.

Figure 18 shows the conformation of the BP-1 molecule and two representative projections of the packing arrangement of the crystal structure. One single molecule represents the asymmetric unit of a two-molecule unit cell of space group P-1. The two phenyl groups are twisted by ϕ = 32.2° (Table 7a). The torsion angles τ1(C13-O1-C1-C2) for tail 1 and τ3(C15-O3-C10-C11) for tail 2 differ, which means the relative placements of the two alkane tail ends are not the same with respect to the planes of the two phenyl groups to which they are attached (*cf.* Table 7a). The projection down the molecular axes suggests that molecular strings of adjacent molecules are present, which can be arranged in various planes. The projection on the a,c-plane shows such two overlapping planes (Figure 18).

**Figure 19:**
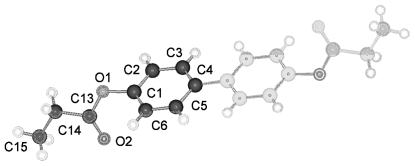

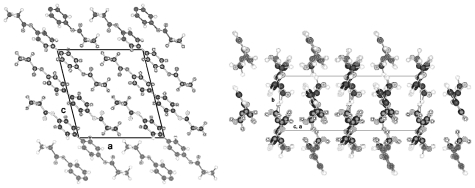
Asymmetric unit of BP-2 (half a molecule) and the corresponding symmetric unit of the molecule. Both halves are connected by an inversion center between the two phenyl rings. Note the two phenyl rings are coplanar. Also represented are the packing arrangements on the a,c-plane and down the molecular strings.

In contrast crystalline BP-2 belongs to space group P2_1_/c with half a molecule as asymmetric unit. The unit cell contains 4 half-molecules or two molecules, which means the same number of molecules in the unit cell as BP-1 in space group P-1. As shown in Figure 19 the symmetry center between the two phenyl rings requires the two phenyl rings to lie coplanar and the two alkane tail groups to exhibit the same twisting angles τ1 and τ3 with respect to the phenyl plane 1 and 2 (*cf.* Scheme 3; Table 7a). These twisting angles are of the same size as τ1 observed for BP-1. However, the twisting angle ϕ between the two phenyl planes changes to 0° for BP-2 as compared to 32.2° for BP-1 and suggests that the packing of the molecules in the unit cell predominantly influences the twisting of the two phenyl rings. The projection on the a,c-plane shows a much clearer picture than for BP-1 inasmuch as the phenyl rings of the molecules form staples. The projection down the molecular axes displays wider spread strings due to the longer side groups in BP-2.

**Figure 20:**
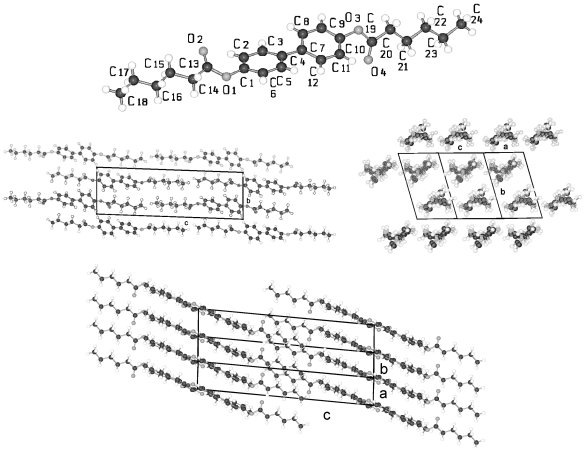
Representation of the asymmetric unit, respectively the molecular conformation of BP-5 with the numbering of the atoms. The planes of the two phenyl rings are twisted by 20.2° against each other. Also represented are the packing arrangements on the b,c-plane, down the molecular strings and along molecular planes.

A single molecule of BP-5 represents the asymmetric unit of a two-molecule unit cell in space group P-1 as for BP-1 and its conformation is shown in Figure 20. Although one of the alkane tails exhibits the same torsion angle τ4 as found for BP-1, called τ2 in this case due to a different numbering scheme, the other torsion angle τ2 for BP-5 towards the alkane tail 1 differs by more than 60° from the corresponding one for BP-1. The twist between the two phenyl planes of BP-5 (ϕ = 20.2°) lies between the ones for BP-1 and BP-2 (*cf.* Table 7a, Scheme 3). Besides the molecular conformation, Figure 20 shows three packing arrangements, one on the b,c-plane that is the projection in [100] direction, in which the molecules forming planes are stapled. Further a projection down the axes of the molecules, which confirms the almost straight running strings despite the deviations from an all-trans conformation of the tail groups. The third projection shows a rotation of the unit cell of the [100] projection around c, which displays the tilt of the molecules and that actually parallel running planes are present.

**Figure 21:**
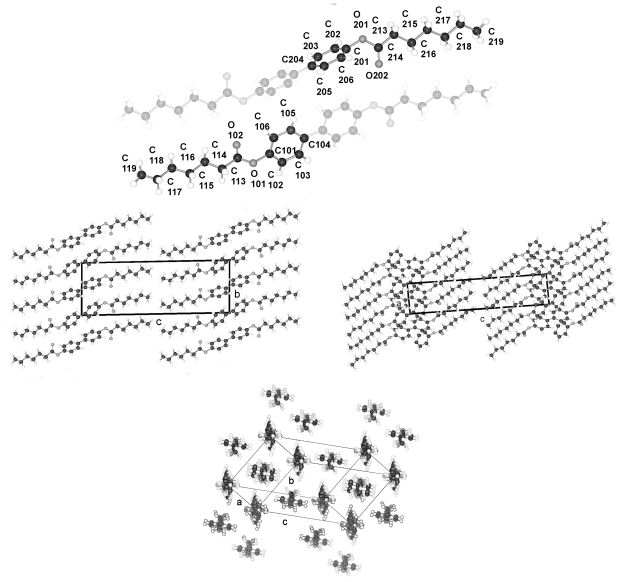
Asymmetric unit of BP-6 (two half-molecules) and the corresponding symmetric units of the molecules linked by an inversion center between the two phenyl rings. Note the two phenyl rings are coplanar. Also represented are the packing arrangements on the a,c-plane, b,c-plane and the projection down the molecular strings. Equivalent representations are obtained for BP-*n*, *n* = 8, 11, 12, 13, 14, if the length of the alkane tails are taken into account.

**Figure 22:**
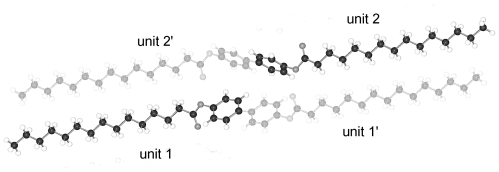
Generation of the two molecules for the structure of BP-14 in space group P-1 by two units 1 and 2 of half a molecule on which inversion symmetry elements are applied.

The structures of BP-*n* (*n* = 6, 8, 11, 12, 13, 14) show equivalent two-molecule unit cells, space group P-1, if the various elongations in c according to the lengths of the molecules are taken into account. As represented in Figure 21 for BP-6 the asymmetric unit consists of two dissimilar half-molecules (Table 7b), unit 1 and unit 2 (*cf.* also Figure 22 for BP-14), which are linked to two single but conformational different molecules by inversion centers. Such an inversion center, but within a single molecule and not within two molecules, is present for BP-2. For BP-6 unit 1 is linked by inversion symmetry to unit 1′ and unit 2 to the inverted unit 2′ that means the torsion angles of unit 1′ and 2′ exhibit opposite signs of unit 1 and 2, respectively. Due to the inversion symmetry within the molecules the adjacent phenyl rings lie coplanar for both molecules that is ϕ = 0 (Table 7b).

Two dissimilar conformations of the two molecules of the series of BP-*n*, *n* = 6 to 14, in the triclinic space group P-1 are present deduced from the different torsion angles τ1(1) and τ1(2) for each of the structures. These values vary by about 20° within the series from *n* = 6 to 14 as do the twisting angles Φ(1) and Φ(2) describing the relationship between the phenyl plane and the plane through the alkane tail (*cf.* Table 7b). The structure refinement suggests that the carbonyl oxygen of unit 1 is in high thermal motion as are the terminal end groups of the alkane chains as well.

The packing arrangement in Figure 21 shown for BP-6 can be extended to all the members of the BP-*n*, *n* from 6 to 14. The molecules of these structures are tilted in three-dimensional space, as represented by the two projection on the b,c- and a,c-plane. The projection down the molecular strings reveals the dissimilar conformation of the two molecules in the unit cell as well as their different rotational position.

The 1,4-di-*n*-alkanoyloxy-biphenyls (BP-*n*) represent an interesting class of symmetrical compounds with a stiff core and flexible tails, which lead to highly ordered smectic phases upon cooling from the melt. Nonetheless, cautious heating of single crystals provide highly oriented crystalline-smectic phases, in which the orientation of the single crystals is preserved as shown for BP-5 by Thyen [9].

All BP-*n* compounds investigated by single crystal structure evaluation exhibit two-molecule triclinic unit cells of space group P-1, except BP-2, which crystallizes in the monoclinic space P2_1_/c but also represents a two-molecule unit cell. The four units of half a molecule for BP-2 in P2_1_/c are linked to two 2 complete molecules by an inversion symmetry center between the two phenyl rings. The asymmetric unit of BP-1 and BP-5 consists of a single molecule, which leads through a space group symmetry element outside the molecules to a two-molecule unit cell. The asymmetric unit of BP-*n* with *n* =6 to 14 consists of two half molecules in the triclinic space group P-1 but here two different single molecules are created through two inversion centers placed between the two adjacent phenyl rings by space group symmetries (Figure 22).

### 2.4. 1,4-Terephthalidene-bis-*N*-(4′-*n*-alkylaniline) (TBAA-*n*)

TBAA-*n* represent by their chemical constitution symmetrical molecules (Scheme 2) as discussed for BP-*n*. However, their packing arrangements in the crystalline state show a much larger diversity than BP-*n* and display a wide variety of space groups and conformations (Table 8). Except TBAA-0 all compounds exhibit liquid crystalline phases: TBAA-*n* with *n* = 1, 2 a nematic phase, with *n* = 3–10 in addition several smectic phases, which have been extensively investigated by Wiegeleben *et al*. [15] and for *n* = 1–6 also by Thyen [9]. The crystal structure of TBAA-4 was determined by Doucet *et al*. [1] who have also studied the transition to the smectic B-phase [11]. Single crystals suitable for a crystal structure determination were grown from solutions of trichloromethane or toluol by solvent evaporation. A summary of basic crystallographic data is listed for TBAA-*n* in Table 8.

TBAA-0 (1,4-terephthalylidene-bis-*N*-(4′-aniline)) crystallizes in the monoclinic space group P2_1_/c with an inversion symmetry center within the molecule and half a molecule as asymmetric unit, which leads to a two-molecule unit cell. For TBAA-1 (1,4-terephthalylidene-bis-*N*-(4′-methylaniline)) space group P2_1_/n was determined, the lateral a and b unit cell dimensions comparable with TBAA-0 but a longer c dimension due to the increase in molecular length. Crystalline TBAA-2 (1,4-terephthalylidene-bis-*N*-(4′-ethylaniline)) displays the orthorhombic space group Pbcn with an inversion center within the molecule that means half a molecule represents the asymmetric unit and the unit cell contains four molecules. One lateral dimension exhibits almost the same magnitude as observed in the previously mentioned structures, the other lateral dimension is doubled and the third dimension adjusted to the length of the molecule (Table 8a). The symmetry center within the central phenyl ring allows a twisting of the two adjacent phenyl rings by the same amount but in opposite directions (Table 9). Such a symmetry center is also observed for the low temperature form of TBAA-5(1) (1,4-terephthalylidene-bis-*N*-(4′-pentylaniline)) at 11 °C. The packing arrangements of the molecules for the further members of the homologous series investigated at ambient temperature exhibit different structures. A whole molecule represents the asymmetric unit with individual not correlated twisting of the three phenyl rings and dissimilar conformations of the two alkyl tails. The monoclinic unit cell, space group P2/a, for TBAA-3 (1,4-terephthalylidene-bis-*N*-(4′-propylaniline)) contains four distorted molecules; the distortion involves mainly one of the two alkyl tails. The same kind of disorder was found for the high temperature form (ambient temperature) of TBAA-5(2) exhibiting the monoclinic space P2_1_/c. TBAA-4 (1,4-terephthalylidene-bis-*N*-(4′-butylaniline)) was investigated by Doucet *et al*. [1] and published as a strongly disordered structure in the monoclinic space group C2/c with 8 molecules in the unit cell. The same space group with comparable unit cell dimensions and similar appearance taking into account the elongation of the molecule was found for TBAA-6 (1,4-terephthalylidene-bis-*N*-(4′-hexylaniline)) without significant disorder of the molecule.

**Figure 23:**
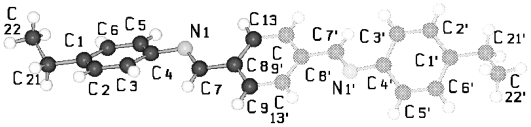
Representation of the conformation of TBAA-2 with an inversion center within phenyl plane 2 (*cf.* Scheme 2) and atom numbering. TBAA-0 and TBAA-1 can be similarly described. Note: a symmetric twisting occurs for the phenyl planes 1→2 with regard to 2→3 for the conformation of the three compounds, but different twisting angles are observed for each of the TBAA-*n* (*n* = 0–2) compound (*cf.* Table 9). The asymmetric unit consists of half a molecule.

Figure 23 shows the conformation of TBAA-2 in the crystalline state at ambient temperature and the numbering of atoms. Since TBAA-0 and TBAA-1 posses also an inversion symmetry element in the central phenyl ring, these structures can be similarly represented. Half a molecule serves as asymmetric unit for all three compounds despite the fact that different space groups and symmetries are present. Single molecules emerge through the inversion at the symmetry center of the centrosymmetric space groups and the number of molecules counts half the formula units in the unit cell.

**Figure 24:**
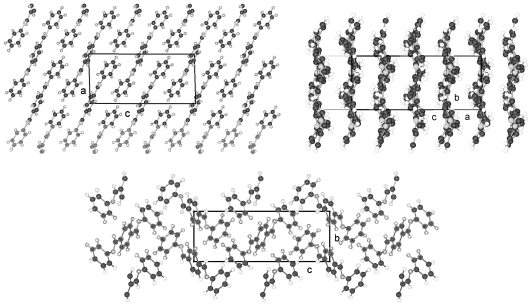
Representation of the packing arrangement of TBAA-0 in three projections on the a,c- and b,c-plane and edge on in the a,c-plane to demonstrate the uniform or respectively the fishbone orientation of the molecules as well as an undulating appearance of the molecular strings.

The packing arrangement of TBAA-0 is represented in three projections in Figure 24. The monoclinic unit cell, space group P2_1_/c with a monoclinic angle β close to 90°, contains 4 formula units or two complete molecules. The molecular axes point in a single direction in a projection on the a,c-plane, in two directions almost perpendicular to each other in the projection on the b,c-plane. Undulated strings result in projection edge on in the a,c-plane due to a 2_1_-screw axis along b by space group symmetry and a tilt of the molecules. These strings in Figure 24 are arranged in wavy planes. A projection down the string axes pointing in one direction occupies a larger area than normally observed for straight chains.

**Figure 25:**
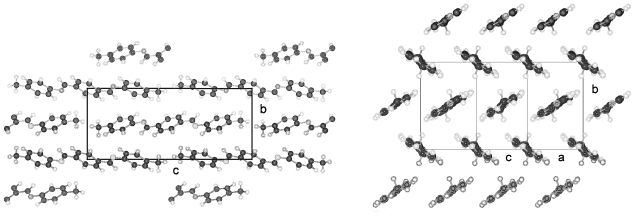
Representation of the packing arrangement of TBAA-1, space group P2_1_/n, in two projections, on the b,c-plane and down the molecular axes to demonstrate the string-like arrangements of neighboring molecules. Note the different orientation of the molecules as compared to TBAA-0 (Figure 24).

In contrast, the molecular axes for TBAA-1 are positioned parallel to the a,c-plane and perpendicular to b (Figure 25) as also found for the structure of POBA shown in Figure 15. Although the symmetry of the space group is the same as for TBAA-0 the packing arrangement exhibits different features, as does the conformation. The three phenyl planes are positioned almost coplanar in contrast to TBAA-0 where the two adjacent phenyl rings are twisted towards the central one by 50° (Table 9) in opposite direction due to the inversion center in the central phenyl ring. The projection down the molecular axes in Figure 25 shows strings arranged in planes.

**Figure 26:**
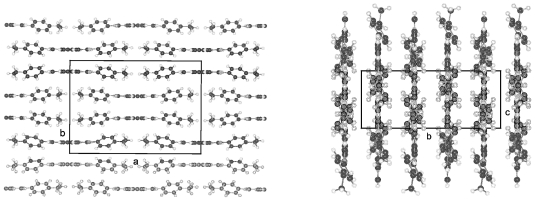
Representation of the packing arrangement of TBAA-2, space group Pbcn, in two projections, on the a,b-plane and down the average molecular axes to demonstrate the string-like arrangements of neighboring molecules. Note the different orientation of the molecules as compared to TBAA-0 (Figure 24) and TBAA-1 (Figure 25).

TBAA-2 crystallizes in the orthorhombic space group Pbcn with 8 formula units or 4 complete molecules in the unit cell. The twisting of the phenyl rings reaches almost the values of TBAA-0 (*cf.* Table 9). The packing arrangement is shown in two projections in Figure 26. The perpendicular placement of the molecular axes towards the b-axis in the a,b-projection is somewhat misleading since the molecules are slightly tilted out of the plane in c- and –c-direction and a further clear projection pattern cannot be provided except the projection on the b,c-plane that means along an average molecular axis. This drawing suggests molecular strings are formed slightly tilted in two directions in the a,c-planes and spread in c-direction as shown in Figure 26.

The extinction of X-ray reflections for the monoclinic unit cell of TBAA-3 suggests space group P2/a. The evaluation of the single crystal structure leads to a distorted conformation with a single molecule as asymmetric unit without an inversion center in the central phenyl ring, which was established as a characteristic feature of the previous discussed structures. Therefore, the unit cell contains 4 molecules.

**Figure 27:**
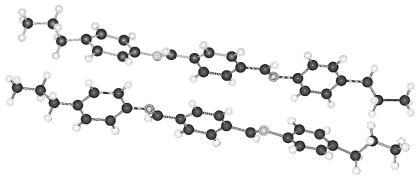
Conformation of TBAA-3 in space group P2 with two independent molecules, representing actually distorted symmetry related molecules in the centrosymmetric space group P2/a.

It was rather difficult to model a disordered structure in space group P2/a with several atoms placed in partial occupancy positions and to describe convincingly the structure by the usual goodness of fit parameters. Therefore, the refinement was successfully carried out with a disturbed structure in space group Pa or more convincingly in P2 and the result summarized in Table 8a. The asymmetric unit in space group P2 should actually consist of two independent single molecules with different twisting angles of the two adjacent phenyl rings towards the central ring (Figure 27). But in the refined structure in space group P2 of TBAA-3 these two twisting angles are equivalent confirming that at least the central part (*i.e*., the three phenyl rings) of the two independent TBAA-3 molecules in space group P2 agree within the assumed accuracy and point actually towards a disturbed space group P2/a. Therefore, the two independent molecules in space group P2 approximate a single molecule asymmetric unit in a four molecule space group P2/a.

**Figure 28:**
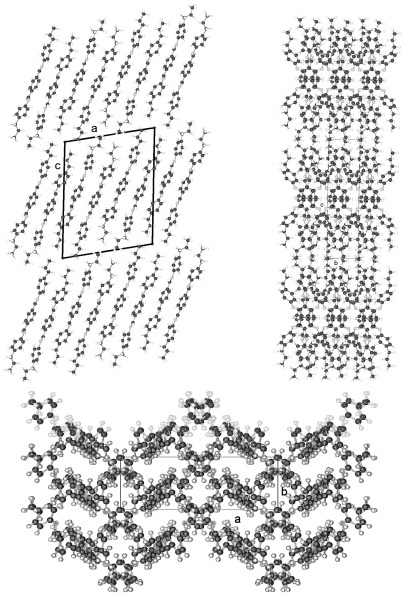
Representation of the packing arrangement of TBAA-3 in three projections on the a,c-plane, on the b,c-plane and on the a,b-plane. Note: a non-parallel arrangement of the molecules as well as the different rotational positions around the molecular axes.

The packing arrangement is represented on the a,c-plane with an uniform orientation of the molecules (Figure 28), which, as shown in the adjacent drawing (b,c-plane), does not represent parallel running molecules in three dimensional space.

TBAA-5 was investigated in two crystalline modifications, one, which exists at 11°C and called TBAA-5(1) and a second one at ambient temperature TBAA-5(2). The two crystalline phases belong to different centrosymmetric space groups, P-1 and P2_1_/c, reflecting the two possible asymmetric units as already discussed, half a molecule with a center of symmetry in the central phenyl ring (plane 2 *cf.* Scheme 2) in space group P-1 and a complete distorted single molecule in space group P2_1_/c with the inversion center outside the molecule.

**Figure 29:**
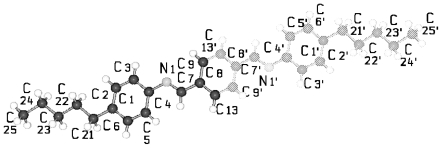
Molecular conformation for TBAA-5(1) in space group P-1 at 11 °C. The phenyl rings lie almost coplanar (as for TBAA-1, Table 9), and also coplanar with the planes through the two alkyl chains.

**Figure 30:**
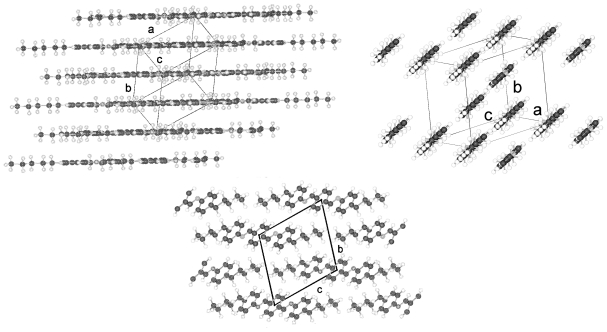
Representation of the packing arrangement of TBAA-5(1) in three projections: One projection shows the molecular planes formed, a second drawing a projection in direction of the molecular axes. Strings can be defined, which can be placed in planes. A third projection in [100] direction represents the stapled and overlapping molecules.

Figure 29 represents the asymmetric unit of half a molecule for crystalline TBAA-5(1) at 11 °C. The complete molecule is generated by application of the inversion symmetry procedure on the asymmetric unit. The three phenyl planes are placed coplanar and lie also coplanar with the planes defined by the two alkyl side groups (*cf.* Table 9). Due to the planar and straight molecular conformation strings can be defined and placed in planes as represented in Figure 30 (top two drawings). The third drawing of Figure 30 shows a projection in [100] direction. The overlapping molecules are stapled and appear as single molecules in the b,c-plane.

**Figure 31:**
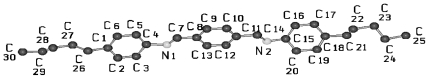
Conformation of crystalline TBAA-5(2) and atom numbering at ambient temperature.

**Figure 32:**
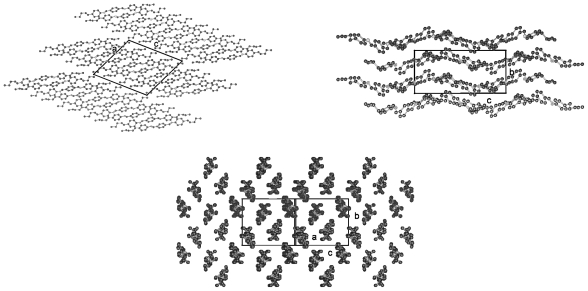
Representation of the distorted packing arrangement of TBAA-5(2) crystallizing in space group P2_1_/c in a,c-projection, b,c-projection and in a projection down the molecular axes.

TBAA-5(2) is formed by a crystalline–crystalline phase transition and represents a different structure than TBAA-5(1). The monoclinic higher symmetric unit cell, space group P2_1_/c, is less densely packed than the one for TBAA-5(1), space group P-1. Table 8b lists for TBAA-5(2) 1.075 g/cm^3^, for TBAA-5(1) 1.144 g/cm^3^. The conformation (Figure 31) deviates considerably from the planar molecule of TBAA-5(1). The phenyl rings are twisted and the alkyl side groups contain disordered gauche conformations. In an overall point of view the conformation is strongly distorted especially the C-atoms in one side group from C27 to C30 (Figure 31) and the positions of these atoms are spread over a wide range. Therefore, the goodness of fit parameter does not exhibit the expected low value. The packing arrangement can be described by a tilted layer structure as occurring in smectic liquid crystals and the molecules forming this structure are lying in undulated planes (Figure 32). Nonetheless, the projection down the molecular axes results in a somewhat spread representation of the molecules as strings.

**Figure 33:**
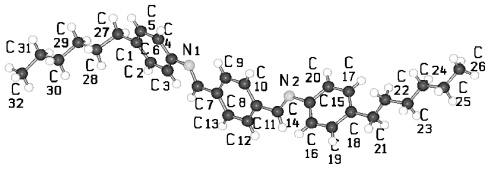
Representation of the conformation and numbering of atoms for TBAA-6 crystallizing in space group C2/c at ambient temperature.

TBAA-6 crystallizes in the highly symmetric C-centered monoclinic space group C2/c with 8 symmetry related molecules in the unit cell. The conformation of a molecule is shown in Figure 33 and exhibits almost the same twist between the phenyl rings as observed for TBAA-3 (Table 9). The twisting of the two alkyl planes towards the phenyl rings to which they are linked displays remarkable differences with 19° for one side group and 69° for the other. The S-shaped molecule does not allow to defining a precise overall molecular axis.

**Figure 34:**
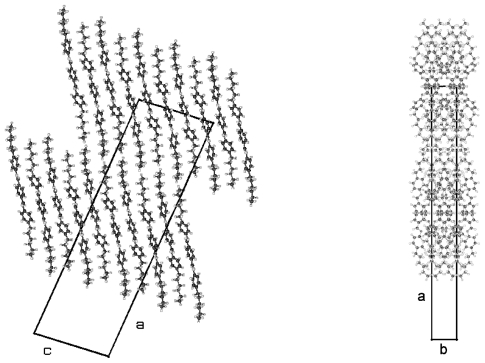
Representation of the packing arrangement of TBAA-6, space group C2/c, in projection on the a,c- and b,c-plane. Note: the similarities with TBAA-3 in Figure 28.

Figure 34 represents the packing arrangement of TBAA-6. The unit cell exhibits a long axis of a = 54.6 Å. Two layers of tilted molecules are positioned along this axis and shown in projection on the a,c-plane. The undulated conformation of Figure 33 appears as a straight molecule in this projection. The molecules are oriented in different directions as shown in projection on the b,c-plane of the adjacent drawing. Comparing TBAA-6 with TBAA-3 it appears that the lateral unit cell dimensions are equivalent with 5.6 Å and 17.4 Å. The long dimensions of both structures are also comparable when adjusting the length of two molecules for TBAA-6 in space group C2/c (two layers) to one molecule for TBAA-3 in space group P21/c (one layer) (Table 8).

A comparison of unit cell dimensions and space groups of TBAA-4 [1] and TBAA-6 (Table 8b) indicates that both structures are closely related, although TBAA-4 was determined as strongly disordered structure. The placement of the unit cell with regard to the molecular orientation seems different comparing the two structures probably due to differences in the monoclinic angle β; the packing arrangement of the molecules (*cf.* Figure 34) appears to be similar in both projections.

## 3. Experimental Section

A CAD4 instrument of Enraf-Nonius was used for the X-ray data collections with a single point detector at ambient temperature, except for TBAA-4 [1] (Philips PW 1100). The structure determination at ambient temperature sometimes led to high motion of the molecules or parts of it in the crystallites, actually intended, since the temperature is then closer to the lc-phase transition temperatures. However, highly accurate investigations are then not possible as provided at low temperatures. The setback of this measurement method includes occasionally distorted structures and a less precise determination of bond lengths and angles, which was anyhow not the goal in this study rather than the investigation was focused on the packing arrangements of the molecules. Most of the single crystals were grown by solvent evaporation of almost saturated solutions. A summary of the basic crystallographic data is listed for each compound. The space groups are determined by selecting the crystal system, searching for systematic extinction of reflections and finally confirming the choice by checking the agreement of observed and calculated intensities for the reflections. The coordinates, bond lengths and angles, torsion angles and further crystallographic data are collected in CIF files and deposited at the Cambridge Crystallographic Data Centre (CCDC 847845-847875).

A survey of further crystals structures of lc mesogen is provided in the publication of Haase and Athanassopoulou [16].

For structure analysis the computer programs from the MolEN package, Enraf-Nonius and for the unit cell refinement CELDIM, Enraf-Nonius [17] was used. The intensity data reduction was carried out with XCAD4NV [18], the structure solution procedure with SIR97 [19], structure refinement with SHELXL-97 [20] and the molecular graphics with SCHAKAL92, 99 [21,22].

## 4. Conclusions

The crystal structures of selected homologous series of rod-like molecules provide valuable information towards modeling crystalline and liquid crystalline phases. The conformation of the molecules is determined to a large extent by the packing interactions. Some of the crystal structures show similarities with smectic liquid crystalline phases as layered structures or intercalated packing arrangements. These crystalline structures may be transformed to smectic phases, but not necessarily to the ones indicated by the crystal structures, since the variability of the conformation and packing is enormous to reach a stable state with lowest free energy.

**Figure 35:**
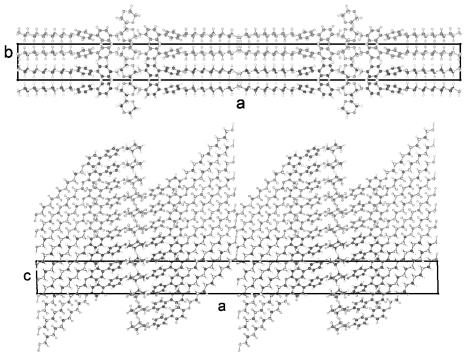
Representation of the crystal structure of H11HBP complexed with 1,4 dioxane in [001] and [010] projection showing a tilted structure such as occurs in smectic liquid crystalline phases. Note the hydrogens of terminal hydroxy groups exhibit partial occupancy both involved in hydrogen bonds.

H11HBP (4-(11-hydroxyundecanyloxy-4′-hydroxybiphenyl) complexed with 1,4-dioxane and shown in Figure 35 (*cf.* also Figure 5 and 6) may serve as an example. The pattern obtained by a projection in [010] direction resembles a tilted smectic phase but with bilayers caused by the hydrogen bonded 1,4-dioxane molecules, which are placed between the H11HBP molecules. The repeat distance amounts to two H11HBP bilayers.

## Figures and Tables

**Table 1:** Summary of crystallographic data for the homologous series of 4-(ω-hydroxyalkyloxy)-4′-hydroxybiphenyl (HnHBP) collected at ambient temperature.

(**a**) HnHBP with *n* = 3, 5, 11; H11HBP complexed with 1,4-dioxane (½ molecule of dioxane in the asymmetric unit)				
	H3HBP	H5HBP	H11HBP	
Molecular formula	C_15_H_16_O_3_	2 × C_17_H_20_O_3_	C_23_H_32_O_3_ × C_2_H_4_O_1_	
Formula mass/g·mol^−1^	244.28	544.66	400.54	
Crystal system	orthorhombic	monoclinic	monoclinic	
Space group/No. Int. Tab.	P2_1_2_1_2_1_/19	P2_1_/c/14	C2/c/15	
a/Å	5.457(1)	15.379(8)	88.30(3)	
b/Å	8.003(1)	5.337(1)	7.1693(9)	
c/Å	28.789(4)	35.11(2)	7.177(2)	
α/°	90	90	90	
β/°	90	99.23(2)	91.47(1)	
γ/°	90	90	90	
V/Å^3^	1257.3(3)	2844(2)	4542(2)	
D_cal_/g·cm^−3^	1.291	1.272	1.172	
Z (formula units)	4	4	8	
CuK_α_ (MoK_α_)/Å	1.54184	0.71069	1.54184	
μ (CuK_α_/MoK_α_)/mm^−1^	0.72	0.086	0.61	
No. of reflections for lattice refinement	16	25	23	
Scan range	22° < θ < 25°	9° < θ < 20°	16° < θ < 43°	
F(000)	520	1168	1744	
Reflections collected	1124	2372	1538	
Unique data	1124 (*R*_int_ = 0.00)	2250 (*R*_int_ = 0.031)	1354 (*R*_int_ = 0.040)	
Significant I’s (>2σ)	1023	1533	1093	
Data collection	3.0° ≤ θ ≤ 60.0°	1.2° ≤ θ ≤ 20.0°	2.0° ≤ θ ≤ 40.0°	
Parameters refined/restraints	168/0	368/0	275/0	
R_1_	0.048	0.051	0.041	
wR_2_	0.136	0.135	0.113	
Goodness-of-fit on F^2^	1.068	1.030	1.100	
Highest peak/e·Å^−3^	0.17	0.23	0.13	
Crystal color	colorless	colorless	colorless	
Crystal size	0.55 × 0.30 × 0.04	platelet	rod-like	
(**b**) HnHBP with *n* = 4, 6, 8, 10; H8HBP evaluated with unit weights using MolEN				
	H4HBP [2]	H6HBP [3]	H8HBP	H10HBP
Molecular formula	2 × C_16_H_18_O_3_	2 × C_18_H_22_O_3_	2 × C_20_H_26_O_3_	2 × C_22_H_30_O_3_
Formula mass/g·mol^−1^	516.61	572.71	628.86	684.92
Crystal system	monoclinic	monoclinic	monoclinic	monoclinic
Space group/No. Int. Tab.	P2_1_/4	P2_1_/4	P2_1_/4	P2_1_/4
a/Å	5.745(3)	5.751(3)	5.705(4)	5.700(1)
b/Å	29.661(9)	34.524(4)	39.18(2)	43.76(1)
c/Å	7.924(4)	7.825(4)	7.764(5)	7.743(1)
α/°	90	90	90	90
β/°	91.04(2)	90.94(3)	90.76(3)	90.770(5)
γ/°	90	90	90	90
V/Å^3^	1350(1)	1553(1)	1735(2)	1931.2(6)
D_cal_/g·cm^−3^	1.271	1.224	1.203	1.178
Z (formula units)	2	2	2	2
CuK_α_ (MoK_α_)/Å	1.54184	1.54184	0.71069	0.71069
μ (CuK_α_/MoK_α_)/mm^−1^	0.70	0.66	0.070	0.076
No. of reflections for lattice refinement	18	25	21	24
Scan range	23° < θ < 25°	19° < θ < 37°	9° < θ < 14°	4° < θ < 11°
F(000)	552	616	680	744
Reflections collected	2263	2191	1569	5532
Unique data	2045 (*R*_int_ = 0.015)	1967 (*R*_int_ = 0.006)	1418	3612 (*R*_int_ = 0.076)
Significant I’s (>2σ)	1959	1888	1083	1801
Data collection	3.0° ≤ θ ≤ 60.0°	2.5° ≤ θ ≤ 54.9°	1° ≤ θ ≤ 20°	1.8° ≤ θ ≤ 20°
Parameters refined/restraints	350/1	386/1	414/1	454/1
R_1_	0.028	0.029	0.066	0.060
wR_2_	0.079	0.079	-	0.145
Goodness-of-fit on F^2^	1.082	1.077	-	1.020
Highest peak/e·Å^−3^	0.12	0.11	0.19	0.18
Crystal color	colorless	colorless	colorless	colorless
Crystal size	0.11 × 0.22 × 0.58	rod-like	platelet	rod-like

**Table 2:** Twist between the planes of different parts of single molecules of HnHBP and between plane 1 of the two molecules, which form the asymmetric units in degrees.

	molecule 1			molecule 2	
	plane 1→plane 2→plane 3		molecule 1→2	plane 1→plane 2→plane 3	
H3HBP	4.6(2)	61.0(3)			
H11HBP	26.4(2)	9.8(3)			
H5HBP	2.4(2)	20.3(3)	66.2(1)	2.5(2)	15.8(4)
H4HBP	3.7(2)	15.8(3)	63.5(1)	11.3(1)	18.7(4)
H6HBP	2.7(2)	15.2(3)	62.5(1)	9.5(2)	18.0(4)
H8HBP	2.9(5)	16.9(17)	61.4(3)	8.2(19)	18.7(19)
H10HBP	2.5(6)	12.3(9)	61.5(3)	8.5(5)	11.8(10)

**Table 3:** Hydrogen bonding of HnHBP.

Hydrogen bonds with H..A < r(A) + 2.000 A and angle (DHA) > 110°.					
D-H	d(D-H)/Å	d(H..A)/Å	<DHA/°	d(D..A)/Å	A
**H3HBP**					
O101-H01	0.820	1.831	163.82	2.629	O103 [x + 1/2, −y + 1/2, −z]
O103-H03	0.820	1.966	166.55	2.770	O101 [−x − 1/2, −y, z − 1/2]
**H11HBP**					
O1-H01	0.887	1.872	172.57	2.754	O101
O3-H03A	0.823	2.072	158.28	2.853	O3 [−x + 1, −y, −z + 1]
O3-H03B	1.383	1.383	154.15	2.696	O3 [−x + 1, y, −z + 3/2]
**H5HBP**					
O101-H011	0.820	1.972	167.95	2.780	O103 [x, −y − 1/2, z − 1/2]
O103-H031	0.820	2.176	159.32	2.957	O101 [x, −y + 1/2, z + 1/2]
O201-H012	0.820	1.988	167.54	2.794	O203 [x, −y + 1/2, z − 1/2]
O203-H032	0.820	2.150	167.57	2.956	O201 [x, −y + 3/2, z + 1/2]
**H4HBP**					
O101-H011	0.820	1.846	166.62	2.651	O203 [−x + 1, y − 1/2, −z]
O103-H031	0.820	1.992	170.52	2.804	O101 [−x, y + 1/2, −z + 1]
O201-H012	0.820	1.868	176.42	2.687	O103 [−x, y − 1/2, −z]
O203-H032	0.820	2.038	155.42	2.804	O201 [−x + 1, y + 1/2, −z]
**H6HBP**					
O101-H011	0.820	1.848	166.81	2.653	O203 [−x + 1, y + 1/2, −z − 1]
O103-H031	0.820	1.955	177.18	2.774	O101 [−x, y − 1/2, −z ]
O201-H012	0.820	1.853	176.39	2.672	O103 [−x, y + 1/2, −z − 1 ]
O203-H032	0.820	1.991	160.28	2.777	O201 [−x + 1, y − 1/2, −z − 1]
**H8HBP**					
O101-H011	0.850	1.786	165.03	2.617	O203 [−x + 1, y − 1/2, −z + 1]
O103-H031	0.850	1.917	173.24	2.764	O101 [−x + 1, y + 1/2, −z + 1]
O201-H012	0.850	1.763	176.06	2.672	O103 [−x + 2, y + 1/2, −z + 1]
O203-H032	0.850	1.918	160.26	2.732	O103 [−x + 2, y + 1/2, −z + 1]
**H10HBP**					
O101-H011	0.820	1.835	160.38	2.622	O203 [−x, y − 1/2, −z]
O103-H031	0.820	2.009	153.71	2.768	O101 [−x + 1, y + 1/2, −z − 1]
O201-H012	0.820	1.840	174.22	2.658	O103 [−x + 1, y − 1/2, −z]
O203-H032	0.820	1.952	170.61	2.765	O201 [−x, y + 1/2, −z]

**Table 4:** Summary of crystallographic data of some 4-substituted benzoic acids collected at ambient temperature.

(**a**) 4-[(*S*)-(−)-2-methylbutoxy]benzoic acid (MBOBA) and racemic (*R*,*S*)-MBOBA as well as 4-(dec-9-enoxy)benzoic acid (DOBA) and 4-(undec-10-enoxy)benzoic acid (UOBA)				
	*S*-MBOBA	(*R*,*S*)-MBOBA	DOBA	UOBA
Molecular formula	2 × C_12_H_16_O_3_	C_12_H_16_O_3_	C_17_H_24_O_3_	C_18_H_26_O_3_
Formula mass/g·mol^−1^	416.50	208.25	276.36	290.39
Crystal system	triclinic	triclinic	monoclinic	triclinic
Space group/No. Int. Tab.	P1/1	P-1/2	P2_1_/n/14	P-1/2
a/Å	7.174(5)	7.107(5)	4.909(2)	4.891(2)
b/Å	9.218(8)	9.421(8)	41.54(1)	8.003(4)
c/Å	9.405(8)	9.518(8)	8.003(4)	22.367(7)
α/°	98.83(4)	96.36(5)	90	94.57(2)
β/°	98.24(4)	100.70(5)	103.93(4)	95.64(2)
γ/°	104.89(4)	109.76(4)	90	102.52(2)
V/Å^3^	583.0(8)	578.9(8)	1584(3)	845.9(6)
D_cal_/g·cm^−3^	1.186	1.195	1.159	1.140
Z (formula units)	1	2	4	2
MoK_α_/Å	0.71069	0.71069	0.71069	0.71069
μ (MoK_α_)/mm^−1^	0.084	0.085	0.078	0.076
No. of reflections for lattice refinement	16	15	23	17
Scan range	7° < θ < 21°	6° < θ < 19°	7° < θ < 19°	7° < θ < 19°
F(000)	224	224	600	316
Reflections collected	1748	1195	1649	1819
Unique data	1748 (*R*_int_ = 0.00)	1078 (*R*_int_ = 0.03)	1441 (*R*_int_ = 0.023)	1569 (*R*_int_ = 0.014)
Significant I’s (>2σ)	1482	491	968	1070
Data collection	2.2° ≤ θ ≤ 23°	2.2° ≤ θ ≤ 20°	2.0° ≤ θ ≤ 20°	1.8° ≤ θ ≤ 20°
Parameters refined/restraints	273/3	138/0	193/6	211/5
R_1_	0.035	0.085	0.058	0.046
wR_2_	0.101	0.207	0.164	0.115
Goodness-of-fit on F^2^	1.080	1.006	1.081	1.040
Highest peak/e·Å^−3^	0.11	0.24	0.30	0.23
Crystal color	colorless	colorless	colorless	colorless
Crystal size	rod-like	rod-like	platelet	platelet
(**b**) 4-(prop-2-enoxy)benzoic acid (PROBA) and 4-(but-3-enoxy)benzoic acid (BOBA) as well as 4-(pent-4-enoxy)benzoic acid (POBA) and 4-(hex-5-enoxy)benzoic acid (HOBA)				
	PROBA	BOBA	POBA	HOBA
Molecular formula	2 × C_10_H_10_O_3_	C_11_H_12_O_3_	2 × C_12_H_14_O_3_	C_13_H_16_O_3_
Formula mass/g·mol^−1^	356.36	192.21	412.46	220.26
Crystal system	monoclinic	monoclinic	monoclinic	monoclinic
Space group/No. Int. Tab.	P2_1_/4	P2_1_/a/14	P2_1_/4	P2_1_/n/14
a/Å	5.147(3)	8.804(7)	10.131(8)	4.779(2)
b/Å	30.50(1)	7.654(2)	7.371(4)	32.34(1)
c/Å	5.717(3)	14.89(1)	15.44(1)	7.993(3)
α/°	90	90	90	90
β/°	95.08(3)	91.58(4)	103.91(6)	101.54(3)
γ/°	90	90	90	90
V/Å^3^	893.9(8)	1003.0(11)	1119(1)	1210.4(8)
D_cal_/g·cm^−3^	1.324	1.273	1.224	1.209
Z (formula units)	2	4	2	4
MoK_α_/Å	0.71069	0.71069	0.71069	0.71069
μ (MoK_α_)/mm^−1^	0.098	0.092	0.087	0.085
No. of reflections for lattice refinement	13	23	21	25
Scan range	10° < θ < 15°	7° < θ < 19°	6° < θ < 11°	18° < θ < 21°
F(000)	376	408	440	472
Reflections collected	1597	1323	1233	2374
Unique data	1481 (*R*_int_ = 0.117)	1221 (*R*_int_ = 0.02)	1149 (*R*_int_ = 0.047)	2114 (*R*_int_ = 0.048)
Significant I’s (> 2σ)	1090	761	785	1211
Data collection	1.3° ≤ θ ≤ 25°	1.4° ≤ θ ≤ 22°	1.4° ≤ θ ≤ 20°	1.2° ≤ θ ≤ 25°
Parameters refined/restraints	238/1	143/5	273/6	159/3
R_1_	0.095	0.055	0.055	0.053
wR_2_	0.324	0.151	0.142	0.136
Goodness-of-fit on F^2^	1.676	1.017	1.226	1.014
Highest peak/e·Å^−3^	0.44	0.26	0.18	0.24
Crystal color	colorless	colorless	colorless	colorless
Crystal size	platelet	platelet	platelet	platelet

**Table 5:** (**a**) Torsion angles τ1, τn in degree describing the placement of the alkoxy group of some 4-benzoic acids: τ1(O3-C7-C8-C9), τn represents the allyl terminal group ending with the double bonded terminal C-atom (partial occupancy: *). Twist angle Φ in degree is defined between phenyl plane and the alkyl plane excluding the double bonded terminal C-atom. (**b**) Torsion angles τ1(1), τn(1), τ1(2), τn(2) in degree describing the placement of the alkoxy group of some 4-benzoic acids: τ1(1)(O103-C107-C108-C109) for molecule 1, τ1(2)(O203-C207-C208-C209) for molecule 2, τn(1), τn(2) represents the allyl terminal group ending with the double bonded terminal C-atom for molecule 1 and 2, respectively. The twist angles Φ(1), Φ(2) in degree are defined between phenyl plane and alkyl plane excluding the double bonded terminal C-atoms for molecule 1 and 2.

(a)				
	UOBA	DOBA	HOBA	BOBA
τ1	67.9 (4)	−67.8 (6)	69.3 (3)	−69.4 (5)
τn *	129.7 (14), −104.6	11 (2), 51.0	−127.3 (14), −60.6	130.6 (12), −134.5 (11)
Φ	68.6 (2)	64.2 (3)	73.2 (2)	70.7 (3)
**(b)**				
	POBA	PROBA	MBOBA(*S*)	MBOBA(*R*,*S*)
τ1(1)	62.7 (13)	135 (2)	59.6 (5)	−47.2 (17)
τn(1)	113 (3)	-	−173.3 (4)	175.0 (11)
τ1(2)	−63.5 (14)	−122 (2)	51.1 (5)	47.2 (17) same as −τ1(1)
τn(2)	−112 (2)	-	−173.3 (3)	−175.0 (11) same as −τn(1)
Φ(1)	67.6 (8)	-	61.4 (3)	51.5 (17)
Φ(2)	63.6 (8)	-	41.2 (2)	51.5 (17) same as Φ1(1)

**Table 6:** Summary of crystallographic data of BP-*n* collected at ambient temperature.

(**a**) 1,4-di-*n*-alkanoyloxy-biphenyl (BP-*n*) with *n* = 1, 2, 5			
	BP-1 [4]	BP-2 [4]	BP-5 [4]
Molecular formula	C_16_H_14_O_4_	2 × C_9_H_9_O_2_	C_24_H_30_O_4_
Formula mass/g·mol^−1^	270.27	298.32	382.48
Crystal system	triclinic	monoclinic	triclinic
Space group/No. Int. Tab.	P-1/2	P2_1_/c/14	P-1/2
a/Å	7.400(6)	11.712(7)	5.505(4)
b/Å	9.227(3)	5.648(1)	8.342(8)
c/Å	10.579(3)	12.408(6)	24.79(2)
α/°	85.97(3)	90	86.67(3)
β/°	89.09(3)	103.840(3)	85.45(6)
γ/°	71.47(6)	90	71.74(7)
V/Å^3^	683.2(6)	797.0(6)	1077.0(16)
D_cal_/g·cm^−3^	1.314	1.243	1.179
Z (formula units)	2	4	2
CuK_α_/Å	1.54184	1.54184	1.54184
μ (CuK_α_)/mm^−1^	0.780	0.714	0.630
No. of reflections for lattice refinement	21	22	21
Scan range	21° < θ < 27°	10° < θ < 21°	17° < θ < 47°
F(000)	284	316	412
Reflections collected	1550	1643	1992
Unique data	1407 (*R*_int_ = 0.0096)	984 (*R*_int_ = 0.047)	1734 (*R*_int_ = 0.0237)
Significant I’s (>2σ)	1288	675	1353
Data collection	4.2° ≤ θ ≤ 50.0°	3.9° ≤ θ ≤ 54.9°	3.6° ≤ θ ≤ 44.9°
Parameters refined/restraints	184/0	103/0	256/0
R_1_	0.045	0.058	0.049
wR_2_	0.121	0.140	0.132
Goodness-of-fit on F^2^	1.038	1.065	1.032
Highest peak/e·Å^−3^	0.23	0.19	0.15
Crystal color	colorless	colorless	colorless
Crystal size	platelet	platelet	platelet
(**b**) 1,4-di-*n*-alkanoyloxy-biphenyl (BP-*n*) with *n* = 6, 8, 11			
	BP-6	BP-8	BP-11
Molecular formula	C_26_H_34_O_4_	C_30_H_42_O_4_	C_36_H_54_O_4_
Formula mass/g·mol^−1^	410.53	466.64	550.79
Crystal system	triclinic	triclinic	triclinic
Space group/No. Int. Tab.	P-1/2	P-1/2	P-1/2
a/Å	5.530(3)	5.426(6)	5.360(3)
b/Å	9.010(8)	9.089(7)	9.249(5)
c/Å	25.19(2)	29.23(3)	34.55(2)
α/°	90.80(5)	91.41(6)	83.55(4)
β/°	91.56(6)	94.85(6)	85.99(4)
γ/°	98.07(5).	98.79(5)	80.16(4)
V/Å^3^	1242.0(16)	1418(2)	1674.7(16)
D_cal_/g·cm^−3^	1.098	1.093	1.092
Z (formula units)	2	2	2
MoK_α_/Å	0.71069	0.71069	0.71069
μ (MoK_α_)/mm^−1^	0.072	0.071	0.069
No. of reflections for lattice refinement	19	21	25
Scan range	5° < θ < 19°	5° < θ < 18°	5° < θ < 11°
F(000)	444	508	604
Reflections collected	1973	2225	5212
Unique data	1709 (*R*_int_ = 0.019)	1940 (*R*_int_ = 0.030)	2673 (*R*_int_ = 0.198)
Significant I’s (>2σ)	1097	894	1260
Data collection	1.6° ≤ θ ≤ 18.0°	2.3° ≤ θ ≤ 18.1°	1.2° ≤ θ ≤ 19.0°
Parameters refined/restraints	274/0	310/0	364/0
R_1_	0.059	0.090	0.095
wR_2_	0.172	0.216	0.237
Goodness-of-fit on F^2^	1.033	1.057	1.065
Highest peak/e·Å^−3^	0.15	0.31	0.23
Crystal color	colorless	colorless	colorless
Crystal size	platelet	platelet	platelet
(**c**) 1,4-di-*n*-alkanoyloxy-biphenyl (BP-*n*) with *n* = 12–14			
	BP-12	BP-13	BP-14 [5]
Molecular formula	C_38_H_58_O_4_	C_40_H_62_O_4_	C_42_H_66_O_4_
Formula mass/g·mol^−1^	578.84	606.90	634.95
Crystal system	triclinic	triclinic	triclinic
Space group/No. Int. Tab.	P-1/2	P-1/2	P-1/2
a/Å	5.429(5)	5.408(4)	5.431(5)
b/Å	9.327(7)	9.327(9)	9.346(8)
c/Å	35.72(4)	37.75(4)	39.17(5)
α/°	84.20(6)	94.52(6)	92.60(2)
β/°	86.81(6)	92.06(6)	90.70(2)
γ/°	79.68(7)	100.27(5)	100.36(8)
V/Å^3^	1769(3)	1865(3)	1953 (3)
D_cal_/g·cm^−3^	1.087	1.081	1.080
Z (formula units)	2	2	2
MoK_α_/Å	0.71069	0.71069	0.71069
μ (MoK_α_)/mm^−1^	0.068	0.067	0.067
No. of reflections for lattice refinement	25	25	15
Scan range	8° < θ < 15°	6° < θ < 18°	6° < θ < 15°
F(000)	636	668	700
Reflections collected	2804	2940	2852
Unique data	2424 (*R*_int_ = 0.038)	2540 (*R*_int_ = 0.046)	2679 (*R*_int_ = 0.006)
Significant I’s (>2σ)	1207	1034	1575
Data collection	1.1° ≤ θ ≤ 18.0°	1.1° ≤ θ ≤ 17.9°	1.0° ≤ θ ≤ 18.0°
Parameters refined/restraints	382/0	400/30	418/7
R_1_	0.055	0.089	0.062
wR_2_	0.138	0.233	0.151
Goodness-of-fit on F^2^	1.018	0.933	1.130
Highest peak/e·Å^−3^	0.16	0.21	0.18
Crystal color	colorless	colorless	colorless
Crystal size	platelet	platelet	0.5 × 0.5 × 0.07

**Table 7:** (**a**) Torsion angles τ1, τ2, τ3, τ4 in degree describing the placement of the tail groups of BP-*n* defined as τ1(C13-O1-C1-C2), τ2(C13-O1-C1-C6) for tail 1, τ3(C(14 + *n*)-O3-C10-C11), τ4(C(14 + *n*)-O3-C10-C9) for tail 2 and twist angles in degree: ϕ between phenyl plane 1 and phenyl plane 2, Φ1 between phenyl plane 1 and alkane plane 3, Φ2 between phenyl plane 2 and alkane plane 4. The torsion angles τ1 and τ2 as well as τ3 and τ4 can be interchanged depending on numbering of the phenyl groups. (**b**) Torsion angles τ1(1), τ2(1), τ1(2), τ2(2) in degree describing the placement of the tail groups of BP-*n* defined as τ1(1)(C113-O101-C101-C102), τ2(1)(C113-O101-C101-C106) for tail 1, τ1(2)(C213-O201-C201-C202), τ2(2)(C213-O201-C201-C206) for tail 2 and twist angles in degree: ϕ between phenyl plane 1 and phenyl plane 2 that is the same for both molecules, Φ(1) between phenyl plane 1 and alkane plane 3 of unit 1, Φ(2) between phenyl plane 1 and alkane plane 3 of unit 2. The torsion angles τ1 and τ2 can be interchanged depending on numbering of the phenyl groups.

(a)						
	BP-1		BP-2		BP-5	
τ1	−121.8 (3)		121.9 (3)		−36.6 (5)	
τ2	61.9 (3)		−65.7 (4)		147.4 (3)	
τ3	−97.4 (3)		as −τ1		−124.2 (4)	
τ4	85.8 (3)		as −τ2		62.5 (5)	
ϕ	32.2 (1)		0.0		20.2 (2)	
Φ1	-		-		18.3 (3)	
Φ2	-		-		56.5 (3)	
(**b**)						
	BP-6	BP-8	BP-11	BP-12	BP-13	BP-14
τ1(1)	−110.0 (7)	−118.0 (12)	−124.9 (9)	−126.3 (7)	−130.0 (10)	127.1 (6)
τ2(1)	77.2 (8)	69.0 (15)	62.2 (12)	61.9 (8)	59.7 (13)	−61.2 (8)
τ1(2)	−100.4 (7)	−106.7 (13)	73.2 (11)	72.2 (8)	71.0 (13)	113.5 (7)
τ2(2)	84.1 (7)	79.8 (13)	−110.8 (11)	−112.2 (7)	−113.1 (11)	−73.4 (8)
ϕ	0	0	0	0	0	0
Φ(1)	61.8 (8)	52.6 (9)	48.9 (5)	39.4 (3)	44.4 (5)	39.8 (3)
Φ(2)	87.4 (5)	83.5 (7)	71.0 (5)	71.6 (3)	68.1 (5)	69.5 (2)

**Table 8:** Summary of crystallographic data of TBAA-*n* collected at ambient temperature.

(**a**) TBAA-*n* with *n* = 0–3. Actual space group P2/a of distorted TBAA-3, refined in P2				
	TBAA-0 [6]	TBAA-1 [6]	TBAA-2 [6]	TBAA-3
Molecular formula	2 × C_10_H_8_N	2 × C_11_H_10_N	C_12_H_12_N	2 × C_26_H_28_N_2_
Formula mass/g·mol^−1^	284.35	312.40	170.23	737.01
Crystal system	monoclinic	monoclinic	orthorhombic	monoclinic
Space group/No. Int. Tab.	P2_1_/c/14	P2_1_/n/14	Pbcn/60	P2/7
a/Å	7.656(3)	6.043(1)	20.9390(2)	17.495(8)
b/Å	6.073(1)	7.8417(4)	14.8707(5)	5.7604(7)
c/Å	16.403(7)	18.215(3)	6.206(1)	22.32(1)
α/°	90	90	90	90
β/°	91.60(2)	92.32(1)	90	100.09(2)
γ/°	90	90	90	90
V/Å^3^	762.4(5)	862.5(2)	1932.4(3)	2215(1)
D_cal_/g·cm^−3^	1.239	1.203	1.170	1.105
Z (formula units)	4	4	8	4
CuK_α_ (MoK_α_)/Å	1.54184	1.54184	1.54184	0.71069
μ (CuK_α_/MoK_α_)/mm^−1^	0.565	0.542	0.521	0.064
No. of reflections for lattice refinement	25	20	25	21
Scan range	10° < θ < 23°	10° < θ < 22°	18° < θ < 25°	7° < θ < 11°
F(000)	300	332	728	792
Reflections collected	1042	1081	995	2835
Unique data	957 *R*_int_ = 0.032	1077 *R*_int_ = 0.18	995 *R*_int_ = 0.00	2716 *R*_int_ = 0.03
Significant I’s (>2σ)	797	822	806	1411
Data collection	5.4° ≤ θ ≤ 55°	4.9° ≤ θ ≤ 55°	3.6° ≤ θ ≤ 50°	1.8° ≤ θ ≤ 22°
Parameters refined/restraints	102/0	112/0	131/0	508/9
R_1_	0.033	0.044	0.050	0.056
wR_2_	0.088	0.125	0.131	0.150
Goodness-of-fit on F^2^	1.099	1.064	1.084	1.003
Highest peak/e·Å^−3^	0.12	0.16	0.15	0.20
Crystal color	yellow	yellow	yellow	yellow
Crystal size	platelet	platelet	platelet	platelet
(**b**) TBAA-*n* with *n* = 4–6, TBAA-4 from [1]; note TBAA-5(1) at 11 °C				
	TBAA-5(1)	TBAA-5(2)	TBAA-4 [1]	TBAA-6
Molecular formula	2 × C_15_H_18_N	C_30_H_36_N_2_	C_28_H_32_N_2_	C_32_H_40_N_2_
Formula mass/g·mol^−1^	424.61	424.61	396.60	452.66
Crystal system	triclinic	monoclinic	monoclinic	monoclinic
Space group/No. Int. Tab.	P-1/2	P2_1_/c/14	C2/c/15	C2/c/15
a/Å	8.394(4)	17.032(7)	53.2(1)	57.58(2)
b/Å	8.915(3)	8.548(2)	5.750(5)	5.634(2)
c/Å	9.038(4)	19.830(8)	17.570(3)	17.402(2)
α/°	103.22(2)	90	90	90
β/°	104.39(2)	114.70(2)	115.47(5)	96.54(2)
γ/°	100.69(2)	90	90	90
V/Å^3^	616.3(5)	2623(2)	4852	5609(3)
D_cal_/g·cm^−3^	1.144	1.075	1.083	1.072
Z (formula units)	1	4	8	8
CuK_α_ (MoK_α_)/Å	0.71069	1.54184	1.54184	0.71069
μ (CuK_α_/MoK_α_)/mm^−1^	0.066	0.468		0.062
No. of reflections for lattice refinement	25	25		17
Scan range	8° < θ < 22°	21° < θ < 25°		8° < θ < 13°
F(000)	230	920	1712	1968
Reflections collected	1639	2214	4020	3132
Unique data	1511 (*R*_int_ = 0.007)	2117 (*R*_int_ = 0.02)	3681	2993 (*R*_int_ = 0.12)
Significant I’s (>2σ)	1112	1381	4020	1630
Data collection	2.4° ≤ θ ≤ 22°	2.9° ≤ θ ≤ 45°		1.4° ≤ θ ≤ 21°
Parameters refined/restraints	149/0	292/8		312/0
R_1_	0.052	0.12	0.133	0.069
wR_2_	0.154	0.36		0.184
Goodness-of-fit on F^2^	1.031	1.683		0.997
Highest peak/e·Å^−3^	0.20	0.53		0.19
Crystal color	yellow	yellow	yellow	yellow
Crystal size	platelet	platelet	0.8 × 0.8 × 0.8	platelet

**Table 9:** Twist between phenyl planes as well as between phenyl plane and alkane plane of TBAA-*n* in degrees (plane assignment *cf.* Scheme 2) at ambient temperature, except TBAA-5(1) at *T* = 11 °C.

plane	1 → 2 → 3 → 4			1→5

n = 0	49.61(8)	same size	-	-

n = 1	2.4(1)	same size	-	-

n = 2	33.57(5)	same size	-	-

n = 3				
(molecule 1)	15.8(9)	52.1(6)		
(molecule 2)	16.9(9)	54.4(6)		

n = 5(1)	1.8(2)	same size	same size as 1→5	1.2(3)

n = 5(2)	15.7(6)	29.2(4)	(alkyl groups: gauche conformation)	

n = 6	16.7(3)	54.6(2)	19.2(13)	69.0(3)
